# Pharmacokinetics of Intrapartum Benzylpenicillin: Insights Into Candidate Regimens to Prevent Early Onset Neonatal Group B *Streptococcus* Disease

**DOI:** 10.1002/psp4.70072

**Published:** 2025-07-08

**Authors:** Bonniface Obura, Jennifer Unsworth, Ana Jimenez‐Valverde, Catriona Waitt, Shampa Das, William Hope, Kate Navaratnam

**Affiliations:** ^1^ Department of Pharmacology and Therapeutics University of Liverpool Liverpool UK; ^2^ Department of Women's and Children's Health University of Liverpool Liverpool UK; ^3^ Infectious Diseases Institute, Makerere University College of Health Sciences Kampala Uganda; ^4^ Fetal Medicine Unit Liverpool Women's Hospital Liverpool UK; ^5^ Harris‐Wellbeing Research Centre University of Liverpool Liverpool UK

**Keywords:** benzylpenicillin, group B *Streptococcus*, neonatal, penicillin, pharmacokinetics, phenoxymethylpenicillin

## Abstract

Early onset neonatal Group B *Streptococcus* (GBS) infection accounts for significant global morbidity and mortality. Intrapartum prophylaxis with benzylpenicillin is advised for women at high risk of having a baby affected by early onset GBS disease. Pregnancy‐related physiological changes can alter pharmacokinetics. To estimate the intrapartum pharmacokinetics of penicillin, women (*n* = 12) at risk of GBS disease were enrolled. A fixed regimen of intravenous benzylpenicillin 3 g at onset of labor and 1.5 g every 4 h until delivery was used. Benzylpenicillin concentrations in plasma and umbilical cord were quantified. A nonparametric population pharmacokinetic model was fitted to the data and regimens that optimized drug exposure (*f*C_min_ > MIC for 100% of the dosing interval) were determined using Monte Carlo simulation. Benzylpenicillin pharmacokinetics were well described using a two‐compartment model with a linked umbilical cord compartment. The mean volume of the central compartment and first‐order clearance were 16.55 L and 41.24 L/h, respectively. Simulations showed that a lower regimen of benzylpenicillin 2.4 g followed by 1.2 g every 4 h resulted in adequate drug exposure—with plasma *f*C_min_ > 0.125 mg/L for 100% of the dosing interval in > 90% of the simulated population. Simulations of a continuous infusion of benzylpenicillin resulted in higher target attainment rates when compared to intermittent dosing. Alternative intrapartum regimens of penicillin that are efficacious but require less total daily drug amounts appear feasible. Further research evaluating alternative regimens on clinical outcomes is required.

**Summary**

- What is the current knowledge on the topic?
  ○ Intrapartum benzylpenicillin is administered to at‐risk pregnant women during labor to prevent the vertical transmission of GBS from mother to child. Pregnancy‐related physiological changes, such as an increase in plasma volume, alter drug pharmacokinetics. Limited evidence exists for regimens that are used for intrapartum prophylaxis.
- What question did this study address?
  ○ This study characterizes the pharmacokinetics of benzylpenicillin in pregnant women during labor and explores alternative regimens that promote improved clinical use and reduced total drug use while maintaining microbiological and clinical efficacy.
- What does this study add to our knowledge?
  ○ Intrapartum benzylpenicillin pharmacokinetics was adequately described by a two‐compartment model, linked to an umbilical cord compartment by first‐order processes. Simulations showed that a lower regimen of benzylpenicillin 2.4 g followed by 1.2 g every 4 h resulted in adequate drug exposure—with plasma *f*C_min_ > 0.125 mg/L for 100% of the dosing interval in > 90% of the simulated population.
- How might this change drug discovery, development, and/or therapeutics?
  ○ We demonstrate that intrapartum benzylpenicillin regimens, which are efficacious but require less total daily drug amounts, are feasible. There is potential to reduce penicillin drug burden during labor.

## 1 Introduction

*Streptococcus agalactiae* (Group B *Streptococcus*; GBS) is a leading cause of severe early onset neonatal sepsis and accounts for significant global morbidity and mortality. The estimated incidence of neonatal sepsis caused by GBS is 0.41 per 1000 live births, with a mortality rate of 4%–10% in high‐income countries [1, 2]. In the United Kingdom, the incidence is rising and is currently 0.57 per 1000 live births [3].

Early onset neonatal GBS disease primarily presents as neonatal sepsis, pneumonia, and/or meningitis and most often occurs 24–72 h postpartum. However, delayed presentations are also seen as late as 6 days after birth. The major risk factor for vertical transmission of GBS from mother to child is GBS colonization of maternal genitourinary and gastrointestinal tracts [4, 5]. Approximately 18% of pregnant women globally are GBS positive, with regional variation of 11%–35% [6]. Transfer of GBS into the uterine cavity during labor plays a vital role in causing GBS disease in term neonates. In preterm neonates, intra‐amniotic infection may be relevant [7]. Additional risk factors include preterm birth (< 37 weeks of gestation), prolonged rupture of membranes ≥ 18 h, extremely low birth weight, intrapartum maternal body temperature ≥ 38°C, GBS bacteriuria, and a previous history of GBS disease [5, 8, 9, 10, 11].

The prevention of GBS disease requires early identification of at‐risk neonates in utero and initiation of prophylactic treatment(s) administered to the mother [12]. Currently, two strategies are used: (1) a risk factor‐based strategy; and (2) a universal screening‐based approach [13]. The risk factor‐based approach considers preterm birth (< 37 weeks of gestation), prolonged rupture of membranes ≥ 18 h, or intrapartum body temperature ≥ 38°C as factors that trigger the administration of intrapartum antibiotics [14]. Alternatively, the universal screening‐based strategy requires that all pregnant women at 35–37 weeks of gestation are sampled for rectovaginal GBS colonization, and a positive result is used to direct the use of antibiotic prophylaxis. In the event of missing GBS culture results at the onset of labor, antibiotic prophylaxis is recommended for those pregnant women with intrapartum risk factors (i.e., prolonged rupture of membranes ≥ 18 h, or body temperature ≥ 38°C) [14]. Both strategies recommend intrapartum antibiotic prophylaxis (IAP) for pregnant women with a history of a baby previously infected by GBS disease, GBS bacteriuria during the current pregnancy, or preterm premature rupture of membranes (< 37 weeks of gestation). A recent systematic review suggests that microbiological sampling approaches result in statistically significantly reduced risk of GBS disease compared with risk‐based strategies (*n* = 17 studies, RR 0·41 [CI 0.30–0.55]) [2].

β‐lactam antibiotics are usually recommended for intrapartum prophylaxis because of their established tolerability, safety, and efficacy against Group B *streptococci* [15]. The β‐lactams display time‐dependent pharmacodynamics—their efficacy is best linked to the fraction of the dosing interval that free drug concentrations are above the minimum inhibitory concentration of the target bacteria (%*f*T>MIC). For established disease, a therapeutic target of at least 40%*f*T>MIC is associated with a favorable clinical outcome [16, 17]. For prophylaxis, pharmacodynamic targets are unknown—for our analyses, we assumed free drug plasma concentrations should be maintained above the MIC of the target organism for the entire dosing interval [18, 19].

Benzylpenicillin is the β‐lactam antibiotic of choice for intrapartum GBS prophylaxis. The National Institute for Health and Care Excellence (NICE) in the United Kingdom and the Royal College of Obstetricians and Gynecologists (RCOG) recommend benzylpenicillin 3 g administered intravenously at the onset of labor, followed by 1.5 g every 4 h until delivery [3]. This regimen requires repeated and regular administration of 30‐min infusions, which can impede mobility in labor and can be challenging to deliver on schedule with other clinical interventions. Moreover, the physiological changes that occur during pregnancy, such as an increase in plasma volume, may alter the pharmacokinetics (PK) of benzylpenicillin [20]. Regimens that are widely cited and used for intrapartum prophylaxis have a limited evidence base. Here, we define the intrapartum pharmacokinetics of benzylpenicillin and use this information to consider alternative regimens that might promote improved clinical use, reduced total drug use to minimize collateral damage to the microbiome and immediate environment, while maintaining microbiological and clinical efficacy.

## 2 Methods

### 2.1 Study Design and Population

A prospective cohort study was conducted at Liverpool Women's Hospital, which is a tertiary level maternal hospital in Liverpool, United Kingdom. Pregnant women ≥ 16 years old, able to understand written and spoken English, in preterm labor (< 37 + 0 weeks gestation), and/or with GBS isolated from an antenatal vaginal swab or urine sample, and/or with a previous baby affected with GBS infection were eligible for inclusion in the study. The exclusion criteria included planned elective caesarean section as antibiotics to prevent early onset GBS disease are not indicated, a history of penicillin allergy, and women with pyrexia (i.e., body temperature ≥ 38°C) during labor as different antibiotics are indicated in these situations. Study participants received the standard IAP regimen of benzylpenicillin, administered as intermittent intravenous infusions over approximately 30 min, with a 3 g loading dose administered at the onset of labor, followed by 1.5 g every 4 h until delivery as per RCOG guidelines [3].

### 2.2 Ethics and Participant Consent

The study protocol was reviewed and approved by the North‐West–Liverpool East Research Ethics Committee (19/NW/0300). Additional approval was granted by the UK's Health Research Authority. The study was compliant with Good Clinical Practice. Written informed consent was obtained from study participants during antenatal visits prior to the onset of labor.

### 2.3 Sample and Data Collection

Participant baseline demographic characteristics (age, weight, and height at booking visit), medical and obstetric history, GBS risk factors, co‐medications, date and time of delivery were recorded. The total number of benzylpenicillin dosages, time of drug administration, and sample collection time were also recorded for each participant. Maternal blood samples of 3 mL were collected in ethylenediaminetetraacetic (EDTA) tubes prior to and at 0.25, 0.5, 1, 2, and 4 h post initial dose. A single umbilical cord blood sample was obtained at delivery. The EDTA tubes containing blood samples were centrifuged at 3000 rpm for 10 min in the Harris‐Wellbeing Research Centre laboratories at the University of Liverpool, and 0.5 mL plasma aliquots were obtained. Plasma samples were immediately stored at −80°C prior to analysis.

### 2.4 Benzylpenicillin Bioanalysis

Benzylpenicillin plasma concentrations were quantified using an ultra‐high pressure liquid chromatography system coupled to a triple‐quadrupole mass spectrometer (LC–MS/MS) (Waters Corporation, Cheshire, UK). The LC–MS/MS assay was validated according to the Food and Drug Administration guidance for industry bioanalytical method validation. The detailed LC–MS/MS methodology is described in the Data S1.

### 2.5 Pharmacokinetic Analysis

The population pharmacokinetics of benzylpenicillin was assessed using the nonparametric adaptive grid (NPAG) algorithm within the Pmetrics package (version 2.1.1; Laboratory of Applied Pharmacokinetics and Bioinformatics, Los Angeles, CA, USA) in R statistical program (version 4.3.1; R Foundation for Statistical Computing, Vienna, Austria) [21, 22]. The NPAG algorithm determines the nonparametric maximum likelihood distribution of parameters in nonlinear modeling [23]. The population parameter distribution generated by NPAG consists of a set of discrete support points, each with a unique combination of parameter estimates and the associated probability of its likelihood [21]. The number of support points is limited to one for each study participant. Population mean parameter estimates are calculated as weighted means, obtained by multiplying each support point's parameter values by its corresponding probability and summing across all points [21]. The standard deviation (SD) of each parameter is derived from its discrete probability distribution. Between‐subject variability, expressed as a coefficient of variation (CV%), is computed for all parameters in the model by dividing parameter SD by the weighted mean.

#### 2.5.1 Structural Pharmacokinetic Model

One‐, two‐, and three‐compartment models with first‐order elimination and intercompartmental transfer were assessed to describe benzylpenicillin maternal plasma and cord blood disposition.

Residual variability between observations and predictions was based on assay error (standard deviation, SD, of observations); where SD was modeled as a polynomial function (SD = C_0_ + C_1_*[observation] + C_2_*[observation]^2^ + C_3_*[observation]^3^). The coefficients C_0_ and C_1_ were estimated as 0.1 and 0.02 respectively from the bioanalytical assay validation data, and C_2_ and C_3_ were fixed at 0. Both proportional error, gamma (residual error = SD × gamma), and additive error, lambda (residual error = [SD^2^ + lamda^2^]^0.5^), were assessed to account for additional process noise related to the observations.

#### 2.5.2 Covariate Modeling

Age, weight (actual total body weight), and creatinine clearance were evaluated as potential covariates. Multivariate regression analysis was used to explore the correlation between covariates and individual pharmacokinetic parameters. Total body weight was recorded during the antenatal visit and includes the actual weight of both the mother and the unborn baby. Creatinine clearance was estimated based on serum creatinine concentrations, age, and weight using the Cockcroft–Gault equation [24]. Covariate effects were assessed using power models with covariates normalized to their median values according to Equation (1).

(1) $$P_{\text{i}}=P_{\text{pop}}\times {\left(\frac{{\text{cov}}_{i}}{{\text{cov}}_{\text{median}}}\right)}^{\alpha }$$

where $P_{\text{i}}$ is the individual parameter value; $P_{\text{pop}}$ is the typical population parameter value; ${\mathrm{cov}}_{i}$ is the individual covariate value, and ${\mathrm{cov}}_{\text{median}}$; is the population median covariate value. Allometric scaling of volume of distribution and clearance was tested using fixed power exponents (α = 1 for volume, and α = 0.75 for clearance) [25].

Covariates were systematically tested in a stepwise approach using forward‐inclusion and backward elimination. A covariate was retained in the model based on physiological plausibility and statistical significance. For forward‐inclusion of covariates, the likelihood ratio test was used to compare two nested models; a change in objective function value (∆OFV) of at least 3.84 was considered significant for retention of a covariate in the model at a significance level of 0.05. Backward elimination of covariates from an intermediate model with all covariates following univariate analysis was informed by statistical significance of 0.001. Comparison of nested models was achieved using the chi‐squared test function within Pmetrics.

Final model selection was based on evaluation of numerical and graphical diagnostics—visual inspection of goodness of fit plots, comparison of OFV, and Akaike information criterion (AIC). In addition, model bias and precision were evaluated using calculated mean weighted error and bias‐adjusted mean weighted squared error respectively—Equations (2) and (3).

(2) $$\text{Bias}\;=\left[\sum \frac{\left(\frac{\text{pred}-\text{obs}}{\mathrm{SD}}\right)}{N}\right]$$

(3) $$\begin{array}{c} \begin{array}{c} \text{Imprecision}\;=\sum \frac{\left[\frac{{\left(\text{pred}-\text{obs}\right)}^{2}}{{\mathrm{SD}}^{2}}\right]}{N}-\sum \frac{\left(\frac{\text{pred}-\text{obs}}{\mathrm{SD}}\right)}{N} \end{array} \end{array}$$

where pred, obs, and SD are the predictions, observations, and standard deviation of observations respectively. *N* is the total number of observations. Model validation and predictive performance were assessed using the simulation‐based visual predictive check.

### 2.6 Target Attainment

Monte Carlo simulation was performed using the simulator in Pmetrics to generate a population of 5000 simulated individuals. A diverse population was simulated by randomly selecting from the distributions of population pharmacokinetic parameters, limiting the simulated values to between 0 and 3 times the maximum observed in the study. The original population covariate‐parameter covariance matrix was retained, that is, mean covariate values of the study population were used for simulation. We evaluated various alternative regimens of benzylpenicillin. The endpoint for therapeutic success was the free drug minimum plasma concentration (*f*C_min_) greater than the MIC for the entire dosing interval (i.e., 100% *f*T>MIC). The target MIC was 0.125 mg/L, which is the epidemiological cut off value for benzylpenicillin against GBS determined from a population of *n* = 3235 GBS samples using EUCAST methodology [26]. The probability of target attainment (PTA) was the proportion of the simulated population with benzylpenicillin free concentrations > 0.125 mg/L for the entire dosing interval in the first 24 h. Target attainment was assessed at 24 h (the longest duration of labor in the study was 20.21 h). Protein binding of 60% was used for all calculations to convert total benzylpenicillin concentration to free concentration [27]. The following regimens were simulated: 3 g, then 1.5 g q4h; 2.4 g, then 1.2 g q4h; 1.2 g q4h; and 1.2 g, then 0.6 g q4h, each administered as a 0.5 h intermittent infusion. Finally, a continuous infusion was assessed.

### 2.7 Cord‐To‐Maternal Plasma Ratio

The extent of benzylpenicillin transfer across the placenta was quantified based on the umbilical cord‐to‐maternal plasma ratio. The area under the concentration‐time curve (AUC_20–24_) of simulated cord and maternal plasma population profiles was compared to derive the cord‐to‐maternal plasma ratio.

## 3 Results

### 3.1 Participant Characteristics

Baseline demographic and clinical characteristics are summarized in Table 1. A total of 12 pregnant women requiring intrapartum benzylpenicillin prophylaxis were included in the study from January 2022 to June 2023. The indications for intrapartum antibiotic prophylaxis included preterm labor (*n* = 5), GBS positive antenatal swab (*n* = 4), and GBS bacteriuria (*n* = 3). All participants received the initial loading dose of 3 g benzylpenicillin. Four participants received a single 1.5 g maintenance dose; three participants received 2 doses, two participants received 3 doses, one participant received 4 doses, and two participants received no maintenance dose. The number of additional doses a participant received was dependent on the duration of labor; there were no protocol deviations. The median (range) maternal age and weight were 30.5 (19–37) years and 75 (52–132) kg, respectively.

**TABLE 1:** Baseline demographic and clinical characteristics of study participants.

Characteristic	Value
Maternal age in years, median (range)	30.5 (19–37)
Weight in kg, median (range)	75 (52–132)
BMI, kg/m^2^, mean ± SD	30.7 ± 10.45
Gravidity, median (range)	2 (1–4)
Parity, median (range)	0 (0–3)
Race	
White, *n* (%)	9 (75)
Asian, *n* (%)	3 (25)
Serum creatinine, mg/dL, mean ± SD	0.56 ± 0.12

### 3.2 Benzylpenicillin Pharmacokinetics

**FIGURE 1:**
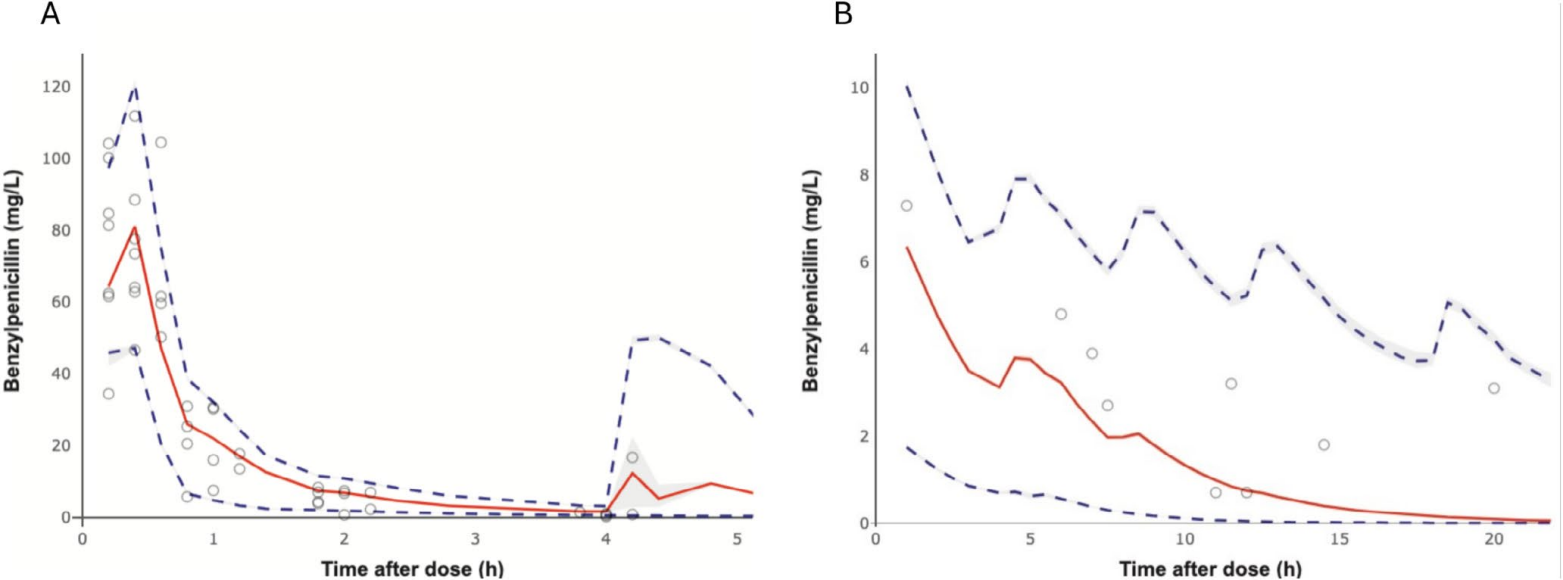
Benzylpenicillin concentration (circles) versus time after dose in maternal plasma (A) and cord blood (B). The red solid line is the median, and dashed blue lines are the 5th and 95th percentiles of population model‐simulated concentration‐time profiles (*n* = 1000). As a visual predictive check, most of the observed concentrations are within 90% prediction interval of the model.

The population pharmacokinetic model was fitted to data from 12 participants with 45 plasma and 10 cord blood observations. A single cord blood concentration was excluded from analysis due to a possible mix‐up of plasma and cord blood samples (same value for plasma and cord blood concentration irrespective of the sampling time post dose). Similarly, 2 plasma samples with unverifiable sampling times were excluded. Benzylpenicillin concentrations in all samples were quantifiable (lower limit of quantification = 0.1 mg/L). Figure 1 shows the distribution of observed penicillin concentrations (circles) in maternal plasma (A) and cord blood (B) superimposed with population model predictions. In maternal plasma, an initial rapid distribution phase occurs in the first hour after administration, followed by a slower elimination phase. Peak plasma concentrations ranged from 31 mg/L to 112 mg/L with a mean ± SD of 75.71 ± 24.02 mg/L. The average area under the concentration‐time curve (AUC_0‐inf_) was 87.88 ± 27.41 mg/L*h using a noncompartmental analysis. The plasma elimination half‐life was 0.54 h. In the umbilical vein, benzylpenicillin concentrations ranged from 0.7 mg/L to 7.3 mg/L.

**FIGURE 2:**
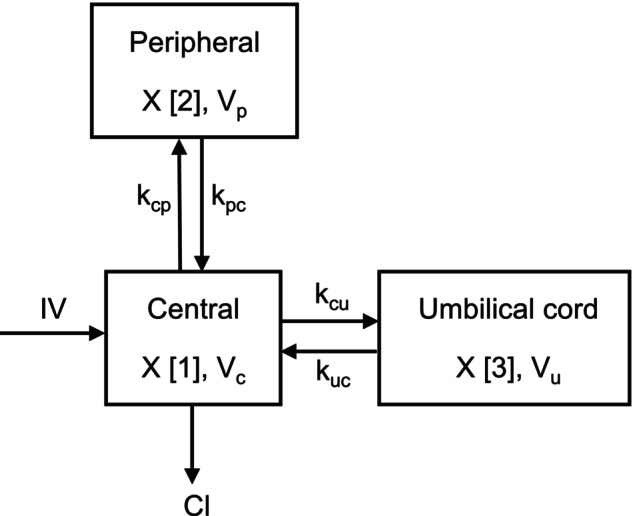
Schematic illustration of benzylpenicillin pharmacokinetic structural model. A two‐compartment plasma pharmacokinetic model of benzylpenicillin, linked to the umbilical cord compartment by first‐order rate constants *k*_cu_ and *k*_uc_. *k*_cp_ and *k*_pc_ are transfer rate constants between the central and peripheral plasma compartments. IV, intravenous infusion; *V*_c_, volume (L) of the central compartment; Cl, clearance (L/h) from the central compartment; *V*_p_ and *V*_u_, volume (L) of the peripheral and umbilical cord compartments respectively.

A two‐compartment model adequately described the observed benzylpenicillin plasma concentrations. A third compartment linked to the central compartment by first‐order rate constants was added to model the observed umbilical cord concentrations. A schematic illustration of the model is shown in Figure 2, and the mathematical implementation is presented in Equations ((4), (5), (6)), which describe the rates of change in amounts of benzylpenicillin.

(4) $$\begin{array}{c} \begin{array}{c} \frac{\mathrm{dX}\left[1\right]}{\mathrm{dt}}=\text{RATEIV}-\left(\frac{\text{Cl}}{V_{\text{c}}}+k_{\text{cp}}+k_{\text{cu}}\right)\times X\left[1\right]+k_{\text{pc}}\times X\left[2\right]+k_{\text{uc}}\times X\left[3\right] \end{array} \end{array}$$

(5) $$\begin{array}{c} \frac{\mathrm{dX}\left[2\right]}{\mathrm{dt}}=k_{\text{cp}}\times X\left[1\right]-k_{\text{pc}}\times X\left[2\right] \end{array}$$

(6) $$\begin{array}{c} \frac{\mathrm{dX}\left[3\right]}{\mathrm{dt}}=k_{\mathrm{cu}}\times X\left[1\right]-k_{\text{uc}}\times X\left[3\right] \end{array}$$

RATEIV is the benzylpenicillin infusion rate into the central compartment of volume, *V*_c_. The amounts of drug in the central, peripheral, and umbilical cord compartments are represented by X [1], X [2] and X [3] respectively. Inter‐compartmental transfer rate constants *k*_cp_, *k*_pc_, *k*_cu_, and *k*_uc_ describe drug transfer to and from the central, peripheral, and umbilical cord compartments. Benzylpenicillin elimination from the central compartment was parameterised as clearance (Cl). Output Equations (7) and (8) describe observed concentrations (mg/L) in the central and umbilical cord compartments respectively.

(7) $$\begin{array}{c} Y\left[1\right]=\frac{X\left[1\right]}{V_{\text{c}}} \end{array}$$

(8) $$\begin{array}{c} Y\left[2\right]=\frac{X\left[3\right]}{V_{\text{u}}} \end{array}$$

**FIGURE 3:**
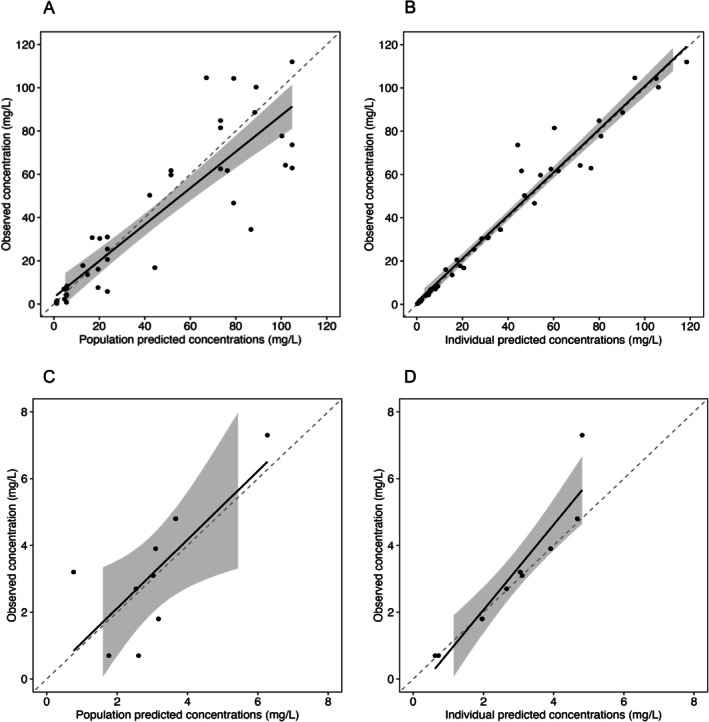
Goodness‐of‐fit plots. Observed versus predicted concentrations: (A) Population predicted plasma benzylpenicillin concentrations. (B) Individual predicted plasma concentrations. (C) Population predicted umbilical cord blood concentrations (D) Individual predicted umbilical cord blood concentrations. Black circles represent observed‐predicted data points, dashed line and solid line represent the identity line and linear regression line respectively. The shaded gray areas represent the 95% confidence interval around the regression lines.

None of the covariates tested—age, total body weight, and creatinine clearance—were significantly related to individual pharmacokinetic parameters; the correlation between weight and volume of central compartment barely approached significance. Adding covariates to the model did not improve the fit of the model to the data or the likelihood in a statistically significant manner after correcting for the additional degrees of freedom. The base model with minimized bias and imprecision was considered the final model. The mean weighted population bias and bias‐adjusted imprecision values were 1.65 and 23.2 respectively for plasma concentrations; 0.125 and 3.79 respectively for cord blood concentrations. A proportional error model best described the residual variability. Plots of observed versus predicted population and individual benzylpenicillin concentrations are shown in Figure 3 and the fit of the model to individual data is presented in Figure S1. A visual predictive check (Figure 1) shows that most observed data lie within the model 90% prediction interval, supporting its use for subsequent simulations. Pharmacokinetic parameter estimates of the final model are shown in Table 2. The coefficients of variation (CV) for clearance and central volume of distribution were 24.06% and 40.15% respectively. This implies that between‐subject variability in volume of distribution was considerably higher compared to clearance.

**TABLE 2:** Population pharmacokinetic parameter estimates.

Parameter (units)	Mean	Median	95% CI	CV (%)
*V*_c_ (L)	16.55	13.69	13.08–20.82	40.15
CL (L/h)	41.24	40.55	32.33–52.3	24.06
*k*_cp_ (h^−1^)	1.49	1.22	0.46–1.94	89.35
*k*_pc_ (h^−1^)	0.98	1.02	0.33–1.5	52.72
*k*_cu_ (h^−1^)	0.09	0.07	0.04–0.13	89.89
*k*_uc_ (h^−1^)	0.5	0.48	0.2–0.84	70.16
*V*_u_ (L)	7.96	7.05	6.2–9.37	51.92
CL ~ *V*_c_ correlation	0.67	—	—	—
Gamma (proportional error model)	4.72	—	—	—

Abbreviations: 95% CI, confidence interval; CL, clearance; CV, coefficient of variation; *k*_cp_, *k*_pc_, *k*_cu_, and *k*_uc_ are first‐order transfer rate constants between maternal central, peripheral, and umbilical cord compartments; *V*_c_, volume of the central compartment; *V*_u_, volume of the umbilical cord compartment.

### 3.3 Simulation and Target‐Attainment Analysis

**FIGURE 4:**
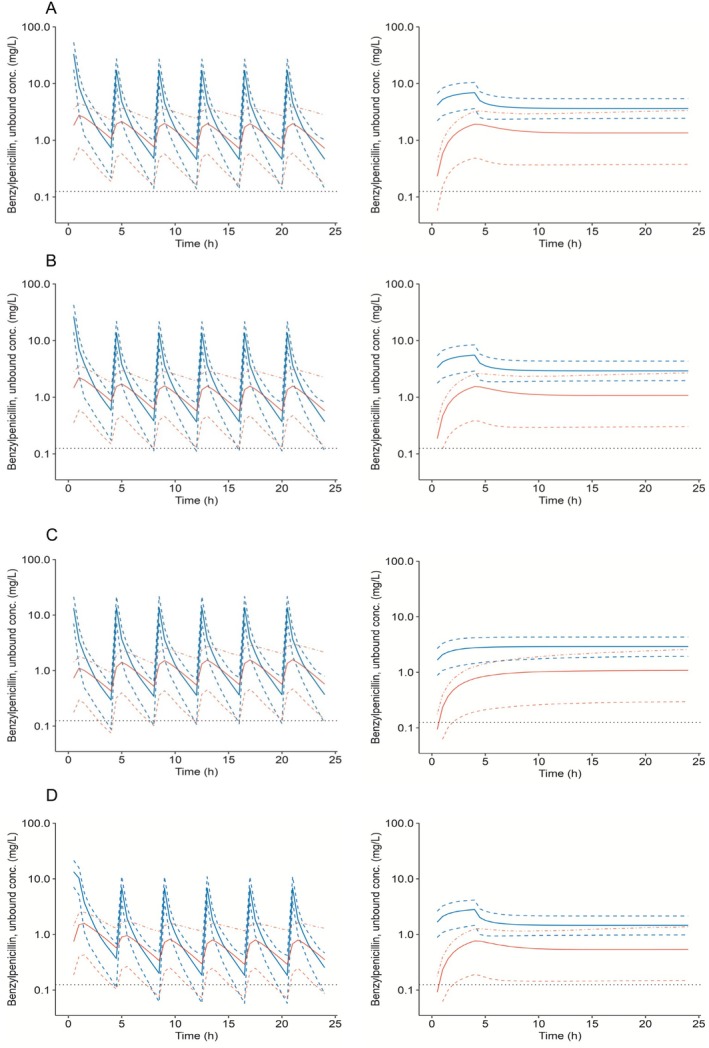
Simulated plasma (blue lines) and cord blood (red lines) concentration‐time profiles for various benzylpenicillin dosing regimens: (A) 3 g, then 1.5 g q4h; (B) 2.4 g, then 1.2 g q4h; (C) 1.2 g q4h; and (D) 1.2 g, then 0.6 g q4h. The regimens were administered as intermittent (left panel) or continuous (right panel) infusions. The solid lines represent the median, and dashed lines are the 5th and 95th percentiles.

Benzylpenicillin concentration‐time profiles for several simulated dosing regimens are shown in Figure 4. Intermittent infusion of the current standard IAP regimen—benzylpenicillin 3 g followed by 1.5 g q4h, as well as an alternative regimen of 2.4 g, then 1.2 g q4h both—exceeded the target MIC of 0.125 mg/L in both plasma and cord blood for 100% of the dosing interval in > 90% of individuals. In contrast, intermittent infusion of benzylpenicillin 1.2 g q4h or 1.2 g followed by 0.6 g q4h did not achieve the pharmacodynamic target in > 90% of individuals. Continuous infusions of the different regimens were effective in maintaining plasma concentrations above 0.125 mg/L for the entire dosing interval. Table 3 shows the proportion of the simulated population with benzylpenicillin maternal plasma and cord blood free concentrations, C_min_ > 0.125 mg/L following dosing with various regimens.

**TABLE 3:** Proportion of the simulated population with benzylpenicillin free concentrations, C_min_ > 0·125 mg/L for various simulated regimens.

Benzylpenicillin dosing regimen	Total daily drug amount (g)	% of individuals with plasma *f*C_min_ > 0.125 mg/L at 24 h	% of individuals with cord blood *f*C_min_ > 0.125 mg/L at 24 h
Intermittent infusion			
3 g, then 1.5 g q4h	10.5	95.82	96.86
2.4 g, then 1.2 g q4h	8.4	92.64	93.92
1.2 g q4h	7.2	88.40	83.5
1.2 g, and 0.6 q4h	4.2	69.16	79.74
Continuous infusion			
3 g, then 1.5 g q4h	10.5	100	74.8
2.4 g, then 1.2 g q4h	8.4	100	68.44
1.2 g q4h	7.2	100	29.06
1.2 g, and 0.6 g q4h	4.2	100	27.94

### 3.4 Benzylpenicillin Cord‐To‐Maternal Plasma Ratio

The mean cord‐to‐maternal plasma ratio of benzylpenicillin was 0.41, with a range of 0.39 to 0.43, following various intermittent infusion regimens. A comparable ratio was observed with continuous infusion regimens, which had a mean of 0.43 and a range of 0.41 to 0.47 (Table S1).

## 4 Discussion

Consistent with previous studies [28, 29], the pharmacokinetics of benzylpenicillin were biphasic (Figure 1). The slower β‐elimination phase in our study population commences approximately 2 h post dose, which is consistent with observations by Johnson et al. [29] in pregnant women during the third trimester of pregnancy. A slow terminal elimination is potentially beneficial for maximizing bacterial killing by exploiting the time‐dependent pharmacodynamics of penicillin [16, 30].

Monte‐Carlo simulations suggest that intermittent infusion of benzylpenicillin 2.4 g followed by 1.2 g every 4 h results in maternal plasma and cord blood C_min_ free concentrations (*f*C_min_) that exceed the target MIC of 0.125 mg/L in > 90% of patients. While the current standard‐of‐care (benzylpenicillin 3 g at onset of labor followed by 1.5 g every 4 h until delivery) achieves adequate drug exposure, similar exposures can be achieved with lower doses of 2.4 g followed by 1.2 g every 4 h. Given that the efficacy of benzylpenicillin depends on the fraction of time that free concentrations remain above the target MIC (*f*T>MIC) rather than the peak concentration achieved, no difference in bacterial killing is expected with use of higher dosages.

Samb et al. [31] suggest that a much lower dose of benzylpenicillin, as recommended by the Dutch obstetrics guidelines—1.2 g followed by 0.6 g every 4 h, might be satisfactory for intrapartum antibiotic prophylaxis. However, simulations of this regimen in our study showed lower than ideal target attainment value (69.16%). This estimate should be qualified by the application of different pharmacodynamic targets. We chose a target defining success as the *f*C_min_>MIC, with the MIC chosen as the epidemiological cutoff value for benzylpenicillin against GBS from EUCAST distributions. This definition of success can be potentially restated as the *f*C_min_>MIC for penicillin susceptible isolates. The pharmacodynamic target for prevention is different from the target for treatment (most often defined as 40%–50% *f*T>MIC). Our estimates and calculations would change in circumstances of emerging penicillin resistance as will undoubtedly occur in the future.

Our results suggest continuous intravenous infusions of penicillin are a highly effective way of achieving the pharmacodynamic target and enable less total daily drug to be used (Table 3). Continuous intravenous infusion of β‐lactam antibiotics is a more efficient dosing strategy than intermittent infusion for increasing the time during the dosing interval that drug plasma free concentrations exceed the MIC (*f*T>MIC) [32, 33, 34]. Whether this advantage, based on modern antimicrobial PK‐PD theory, translates into comparable clinical outcomes requires appropriately designed and powered clinical studies. Rather than seeking improved clinical outcomes as per the randomized controlled studies conducted in critically ill patients by the Beta‐Lactam Infusion Group (BLING studies) [35, 36], a reasonable strategy would be to ensure that regimens that are significantly easier to administer and require less total daily drug are at least noninferior to currently recommended regimens. Optimal dosing strategies of intrapartum penicillin that utilize low total daily drug per patient can minimize disruption of the maternal microbiome and its immediate environment, slow the development of antimicrobial resistance, thus preserving the efficacy of antibiotics.

Transplacental transfer of benzylpenicillin was expressed as a cord‐to‐maternal plasma ratio, with mean values of 0.41 and 0.43 following simulation of various intermittent and continuous infusion regimens, respectively. In the literature, fetal‐to‐maternal ratios are reported to range from 0.3 to 0.9 for penicillin, ampicillin, cefotaxime, cefuroxime, metronidazole, and clindamycin [37]. Several factors influence the transplacental transfer of drugs, most notably: placental permeability, protein binding, ionization, and fetal and maternal clearances [38]. The interrelationship between these factors forms a basis for understanding the observed cord‐to‐maternal plasma ratio. The relatively low cord‐to‐maternal plasma ratio suggests less rapid placental distribution of benzylpenicillin and is attributable to the rapid elimination from maternal plasma.

The therapeutic success of intrapartum antibiotic prophylaxis is based on achieving adequate antibiotic exposure to reduce vertical transmission of GBS and prevent GBS infection in the newborn [39]. In our study, all observed maternal plasma and cord blood concentrations were above the target MIC of 0.125 mg/L. Evidence suggests that cord blood concentrations of penicillin rise rapidly and exceed the target MIC by 10 to 179‐fold, while amniotic fluid levels increase gradually following maternal drug administration [40, 41]. Barber et al. [40] demonstrated that cord blood levels of benzylpenicillin peak at around 1 h, declining rapidly thereafter, following a maternal 3 g loading dose.

The available literature on intrapartum benzylpenicillin pharmacokinetics is largely based on noncompartmental analysis and regression methods used for comparison of maternal plasma and umbilical cord concentrations [29, 31, 40, 42]. Given that assessment of fetal drug exposure is often limited to a single cord blood sample drawn at the time of birth, regression analysis of maternal plasma and cord blood concentrations provides little information on drug exposure over time [43]. Our study investigates intrapartum benzylpenicillin disposition using a population pharmacokinetic approach, and simulations were performed to assess target attainment and cord‐to‐maternal exposure.

The current study has several limitations. We were only able to study a relatively small number of pregnant women; however, the sample size is adequate for providing initial estimates of the population pharmacokinetics. A larger sample size would enable more robust estimates of central tendency and dispersion of the population pharmacokinetic parameters. Secondly, fetal drug exposure was assessed using a single cord blood sample per subject, which contributed to a large between‐subject variability in pharmacokinetic parameter estimates. The single sample per subject sampling approach is valuable for conducting PK studies in populations where intensive sampling is impractical. However, a well‐defined sampling schedule is required to yield more precise population parameter estimates. In our study, the timing of cord blood sampling was dependent on the time of childbirth, and this varied widely across individuals. Third, evaluation of total body weight as a covariate was limited by the inability to differentiate between maternal and fetal weight contributions. Alternative body size metrics such as lean body weight or adjusted body weight were not considered due to similar limitations. Lastly, we assumed a constant unbound fraction of benzylpenicillin for the analysis of target attainment. Given that alteration in benzylpenicillin protein binding in late pregnancy is minimal, we do not anticipate this assumption of a constant unbound fraction to significantly impact our conclusions.

In summary, alternative intrapartum regimens of penicillin that are easier to use and require less total daily drug appear feasible. Our analyses provide the first critical step to considering further research to address the prevention of GBS infections.

## Author Contributions

B.O., C.W., K.N., and W.H. wrote the manuscript. K.N. and W.H. designed the study. J.U., A.J.‐V., and K.N. performed the research. B.O., S.D., and W.H. analyzed the data.

## Conflicts of Interest

W.H. holds or has recently held research grants with UKRI, EU (FP7, IMI‐1, IMI‐2), Wellcome, F2G, Spero Therapeutics, Antabio, Pfizer, Allecra, Bugworks, Phico Therapeutics, BioVersys, Global Antimicrobial Research and Development Partnership (GARDP) and NAEJA‐RGM. He is (or has recently been) a consultant for Appili Therapeutics, F2G, Spero Therapeutics, Pfizer, GSK, Phico Therapeutics, Pulmocide, and Mundipharma Research Ltd. He was a member of the Specialist Advisory Committee for GARDP (2020–2023), a member of the British Society for Antimicrobial Chemotherapy (BSAC) Breakpoint Committee (2020–2023), a member of Health Technology Appraisal (HTA) Prioritization Committee for hospital care and is the Specialty National Co‐lead for Infection for the National Institute of Health Research (NIHR) (2020‐current). C.W. is supported by Wellcome Trust Clinical Research Career Development Fellowship 222075_Z_20_Z. All the other authors declare no competing interests.

## Supporting information


Figure S1.

Table S1.

Data S1.
