# Supplementary material for: Pharmacokinetics of Intrapartum Benzylpenicillin: Insights Into Candidate Regimens to Prevent Early Onset Neonatal Group B Streptococcus Disease
Source: CPT Pharmacometrics Syst Pharmacol. 2025 Jul 8;14(9):1504–14. doi: 10.1002/psp4.70072 (PMC12439288; doi:10.1002/psp4.70072)

**Figure S1:** Plots of individual model fit. The red and blue circles are the observed benzylpenicillin concentrations, while the red and blue lines are the model predictions in plasma and cord blood respectively.


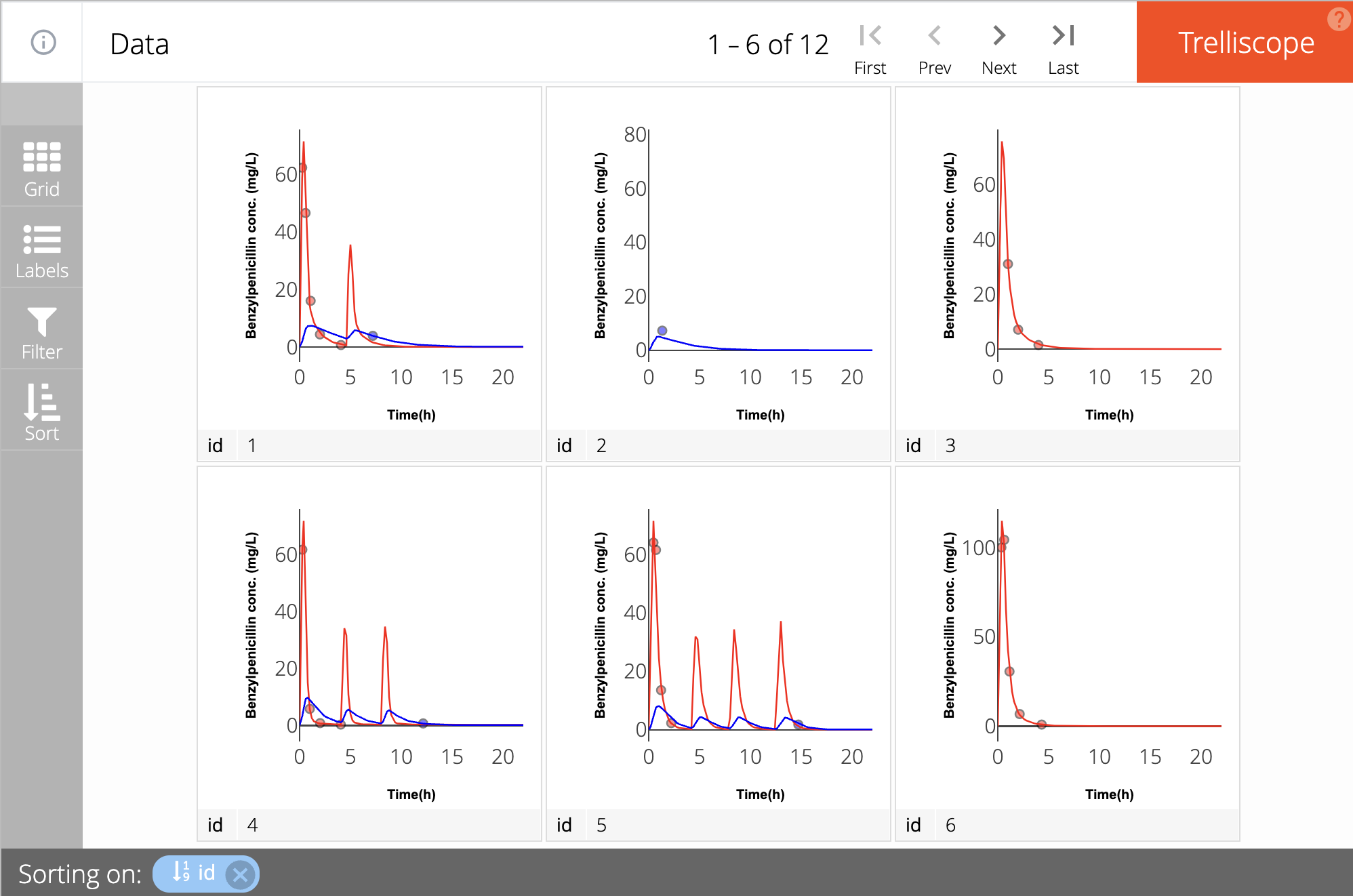

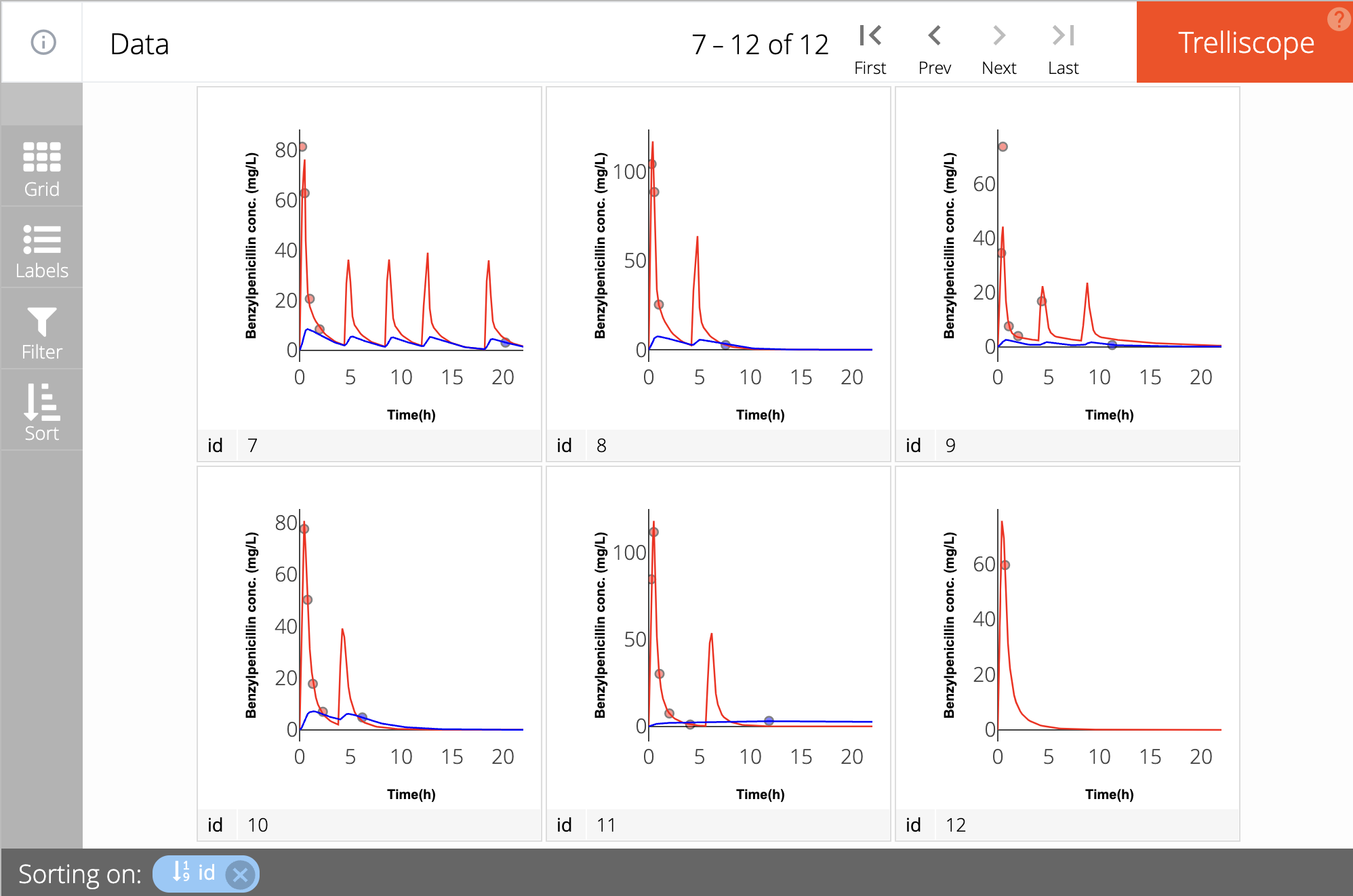

Supplement: Supplementary file 1 — Figure S1. [file PSP4-14-1504-s001.docx]
