# Supplementary material for: Pharmacokinetics of Intrapartum Benzylpenicillin: Insights Into Candidate Regimens to Prevent Early Onset Neonatal Group B Streptococcus Disease
Source: CPT Pharmacometrics Syst Pharmacol. 2025 Jul 8;14(9):1504–14. doi: 10.1002/psp4.70072 (PMC12439288; doi:10.1002/psp4.70072)
Supplement: Supplementary file 2 — Table S1. [file PSP4-14-1504-s003.docx]

**Table S1:** Simulated maternal plasma and cord blood benzylpenicillin area under concentration-time curve (AUC)

| Benzylpenicillin dosing regimen | | Mean AUC_20-24_ (mg.h/L) | | Cord to plasma ratio |
| --- | --- | --- | --- | --- |
|  |  | Maternal plasma | Cord blood |  |
| 3g, then 1.5g q4h | Intermittent dosing | 14.91 | 6.07 | 0.41 |
|  | Continuous infusion | 14.87 | 6.08 | 0.41 |
| 2.4g, then 1.2g q4h | Intermittent dosing | 11.93 | 4.85 | 0.41 |
|  | Continuous infusion | 11.89 | 4.87 | 0.41 |
| 1.2g q4h | Intermittent dosing | 11.90 | 4.74 | 0.39 |
|  | Continuous infusion | 11.88 | 5.57 | 0.47 |
| 1.2g, then 0.6g q4h | Intermittent dosing | 5.99 | 2.56 | 0.43 |
|  | Continuous infusion | 5.94 | 2.63 | 0.44 |
