# Supplementary material for: Pharmacokinetics of Intrapartum Benzylpenicillin: Insights Into Candidate Regimens to Prevent Early Onset Neonatal Group B Streptococcus Disease
Source: CPT Pharmacometrics Syst Pharmacol. 2025 Jul 8;14(9):1504–14. doi: 10.1002/psp4.70072 (PMC12439288; doi:10.1002/psp4.70072)
Supplement: Supplementary file 3 — Data S1. [file PSP4-14-1504-s002.docx]

**Pharmacokinetics of intrapartum benzylpenicillin: insights into candidate regimens to prevent early onset neonatal group B *Streptococcus* disease – Supplementary material**

**Text S1:** **Benzylpenicillin bioanalysis method**

Benzylpenicillin plasma concentrations were quantified using an ultra-high pressure liquid chromatography system coupled to a triple-quadrupole mass spectrometer (LC-MS/MS) (Waters Corporation, Cheshire, UK).

Sample preparation and extraction was achieved using protein precipitation. The internal standard, ^2^H_7_ benzylpenicillin (Alsachim, France) was prepared mixed with acetonitrile (Fisher Scientific UK) and 150 μL was added to a 96-well protein precipitation plate (Phenomenex, Cheshire, UK). A total of 50 μL each of samples, blanks, calibrators in the range 0.1 – 100 mg/L and quality controls were mixed with the internal standard on an orbital shaker for 5 mins at 800 rpm. A positive pressure manifold was used to filter liquid through the protein precipitation plate into a collection plate with water and acetonitrile containing 0.1% formic acid (200 μL) added to each well. Subsequently, the plates were sealed, and mixing was performed on an orbital shaker prior to LC-MS/MS analysis.

Chromatographic separation was achieved by injecting 2 μL of analytes onto an ACQUITY Premier HSS T3 Column (100A, 1.8 μm, 2.1 mm X 100 mm) and separation over a 4.5 min gradient using a mixture of solvents A (LC-MS grade water + 0.1% formic acid) and B (LC-MS grade acetonitrile + 0.1% formic acid). Separations were accomplished by applying a linear gradient of 95% to 5% solvent A over 3.5 mins at 0.4 mL/min followed by equilibration for 1 min at 95% solvent A.

The mass spectrometer was operated in negative ion mode. Multiple Reaction Monitoring (MRM) method was used with the specified mass transitions and collision energies: benzylpenicillin 332.94 > 73.8 (Ce 22 eV); 332.94 > 191.81 (Ce 10 eV), and ^2^H_7_ benzylpenicillin 339.99 > 171.89 (Ce 16 eV). Mass spectrometry readouts were processed using MassLynx MS software. The mass spectrometer conditions were as follows: capillary voltage of 2.5 kV; desolvation temperature 600^o^C; desolvation gas flow 1000 L/h; source gas temperature of 150^o^C.  Data were processed using TargetLynx XS software. The analytical method was validated to assess recovery and matrix effects, carryover, inter-day and intra-day accuracy and precision. The mean recovery from matrix was 89.73% and lower limit of quantification (LLOQ) was 0.1 mg/L. The inter- and intra-day %CV on the three QC levels ranged from 7.22% – 10.44% and 3.14% – 5.44% respectively.

**Text S2: Pmetrics model file**

#Primary variables

Vc, 0.00, 40.00 ; volume of the plasma central compartment

Cl, 0.00, 60.00 ; clearance from central compartment

K12, 0.00, 4.00 ; first-order transfer rate constant from central to peripheral compartment

K21, 0.00, 1.50 ; first-order transfer rate constant from peripheral to central compartment

K13, 0.00, 0.50 ; first-order transfer rate constant from central to the umbilical cord

K31, 0.00, 1.00 ; first-order transfer rate constant from umbilical cord to the central

Vu, 0.00, 20.00 ; volume of umbilical cord compartment

#Covariates

age

weight

Crcl

#Secondary variable

Ke = Cl/Vc

#Equations

dX[1] = RATEIV[1] - (Ke+K12+K13)*X[1] + K21*X[2] + K31*X[3]

dX[2] = K12*X[1] - K21*X[2]

dX[3] = K13*X[1] - K31*X[3]

#Outputs

Y[1]=X[1]/Vc

Y[2]=X[3]/Vu

#Error

G=10.00 ; gamma – multiplicative error

0.10, 0.02, 0.00, 0.00 ; coefficients for the polynomial equation for plasma concentrations

0.10, 0.02, 0.00, 0.00 ; coefficients for the polynomial equation for cord blood concentrations
